# CT findings of severe novel coronavirus disease (COVID-19): A case report of Heilongjiang Province, China

**DOI:** 10.1515/med-2020-0151

**Published:** 2020-06-13

**Authors:** Ziao Wang, Huijie Jiang, Hao Jiang, Ruoshui Zheng, Ru Yi

**Affiliations:** Department of Radiology, The Second Affiliated Hospital of Harbin Medical University, Heilongjiang Province, China

**Keywords:** COVID-19, SARS-CoV-2, novel coronavirus pneumonia, CT findings, SARS-CoV-2 pneumonia

## Abstract

2019 novel coronavirus disease (COVID-19, previously known as novel coronavirus pneumonia) was first discovered in December 2019 and spread widely in China and all over the world in 2020. The initial symptoms of most patients include fever, cough, and fatigue. Dyspnea may occur with the progress of the disease, and acute respiratory distress syndrome may occur in severe cases. The CT manifestations of this disease are mainly ground-glass opacity (GGO) in the lung, which may be accompanied by patchy consolidation, and fibrous changes may appear in the lung at the later stage of the disease. Combined with typical clinical and imaging findings and positive nucleic acid test results, the disease can be diagnosed. We report the first case of novel coronavirus disease (COVID-19) in Heilongjiang Province, China. The patient was seriously ill, who felt that he suffered from fever, fatigue, cough, and expectoration and sought medical treatment, with a history of contact with Wuhan. The leukocyte count was normal, and the lymphocyte count was decreased. CT imaging showed large GGO and partial patchy consolidation in both lungs. The patient recovered and was discharged after 26 days of treatment. This study is helpful for early diagnosis and timely clinical management by mastering the typical imaging of novel coronavirus disease (COVID-19).

## 1 Introduction

Novel coronavirus disease (COVID-19, previously known as novel coronavirus pneumonia [NCP]) is an acute pulmonary inflammation caused by severe acute respiratory syndrome coronavirus 2 (SARS-CoV-2) infection. In December 2019, several cases with pneumonia caused by unknown reasons occurred in Hubei Province, China. Most of these patients worked or lived nearby the local Huanan China Seafood Wholesale Market. Some of them rapidly developed acute respiratory distress syndrome (ARDS) and other complications after a few days of onset. In January 2020, the Chinese Center for Disease Control and Prevention found and confirmed the virus for the first time, which is known as SARS-CoV-2. In the next several months, the disease spread widely in China and other countries, infecting more than 2,00,000 people. On 30 January 2020, WHO declared the outbreak of COVID-19 as a public health emergency of international concern. In this article, the first case with severe NCP in Heilongjiang Province, China, was reported, aiming to state related knowledge for reference by colleagues, improve the understanding for imaging manifestations of COVID-19, facilitate the early diagnosis of this disease, and guide the next clinical decision-making.

## 2 Case details

A 69-year-old man who suffered from fever, fatigue, myalgia, cough, and expectoration went to the hospital for medical treatment; temperature at admission: 38.2 ℃; heart rate: 78 times/min; respiration: 22 times/min; and blood pressure: 142/75 mm Hg. The patient stated that he had traveled back to Harbin from Wuhan, Hubei Province, by aircraft 1 day before and returned to Mudanjiang city, Heilongjiang Province from Harbin by high-speed railway. The patient stated that he had a smoking history, no drinking history, and suffered hypertension. The results of laboratory examination after admission are as follows: normal leucocyte count (6.17 × 10^9^/L), decreased lymphocyte count (0.72 × 10^9^/L), increased C-reactive protein (64.5 mg/L), increased erythrocyte sedimentation rate (28 mm/h), and increased D-dimer (1.44 µg/mL). CT examination showed that the two lungs had a large area of ground-glass opacity (GGO) and a small area of patchy consolidation. The lesions had an obscure boundary, which distributed mainly in a subpleural area. CT images also reveal “air bronchography sign,” enlargement of mediastinal lymph nodes, and bilateral pleura thickening. The patient was admitted to the hospital for antiviral treatment and symptomatic supportive treatment.

Two days after admission, the patient developed dyspnea. Because the patient was seriously ill, he was admitted to the ICU for the treatment and received tracheal cannula and continuously underwent symptomatic supportive treatment, antiviral treatment, and prophylactic antibacterial treatment. After 9 days of admission, the nucleic acid test showed that the result of SARS-CoV-2 was positive, and the patient was diagnosed with COVID-19, who was the first case in Heilongjiang Province, which was imported. During the period of hospitalization, the general situation of the patients was relatively stable. After 16 days of admission, CT examination was performed again: large areas of GGO and consolidation were still seen in both lungs, mainly distributed in the lower lobes of both lungs. However, compared with the previous images, the area of GGO was reduced and more tended to be distributed in subpleural. The area of patchy consolidation increased slightly. The grid shadow appeared near the pleura of both lungs, showing the fibrous change.

The condition of the patient gradually improved. On the 20th day after admission, the reexamination of CT showed that the areas of GGO and consolidation shadow in both lungs were significantly reduced, and the lesions were absorbed. There were more grid shadows in both lungs, main subpleural distribution, and fibrous changes in both lungs. The patient continued to receive supportive treatment. The patient was generally in good condition with normal body temperature, and the laboratory examination indicators gradually returned to normal. Then, the patient presented the pathogen test of SARS-CoV-2 RNA twice, and the results were all negative. The patient was discharged on the 26th day after admission and was clinically cured.

## 3 Discussion

Since the discovery of the 2019 novel coronavirus disease (COVID-19) in Wuhan, Hubei Province, in December 2019, the number of cases has increased rapidly, showing a spreading trend in China and other countries overseas. By 20:00 on 21 March 2020, there were 81,457 confirmed cases and 3,261 dead cases in China, and 2,01,577 cases were confirmed in overseas areas, involving more than 100 countries. COVID-19 has become pandemic all over the world. For the first visit, the common clinical manifestations of patients showed fever, weakness, dry cough, and myalgia, while some patients showed headache, abdominal pain, diarrhea, and nausea for the first time. With the progress of the disease, some patients suffered dyspnea about 1 week after admission. Severe patients might have shock, arrhythmia, and ARDS [1].

Imaging examination is an important method to diagnose COVID-19. Chest radiography shows a patchy shadow in the lung field, which is a preliminary judgment of the disease. However, chest radiography has a low diagnostic rate for small lesions and is not the first choice because of the blocking effect of mediastinal shadow. We can find lesions from the tomography and carry out three-dimensional reconstruction by CT examination to diagnose COVID-19 more accurately. It can accurately assess the size, distribution, involved range, and severity of the lesion. Therefore, the CT examination plays a crucial role in the diagnosis of NCP.

The main CT imaging feature of COVID-19 includes bilateral GGO, mainly with the subpleural distribution. The imaging of mild cases shows the focal distribution. CT findings of severe cases show the diffuse distribution in double lung fields such as the case we reported. GGO can be accompanied by consolidation, which is mainly seen in critically ill patients with “white lung” changes [2]. “Air bronchography sign,” thickened vascular shadow were seen in lesions. Thickened interlobular septa and “crazy-paving pattern” can also be seen in CT imaging [3]. Because the patient we reported is under the severe condition, lesions on the CT are quite obvious. CT imaging shows large areas of GGO, which is a classical manifestation. With the development of the disease, fibrous changes can be formed, which is manifested as a grid shadow. Fibrous changes illustrate the destruction of lung tissues, especially in severe cases. In the third CT examination of our patient, fibrous stripes exist definitely. Some patients have enlarged mediastinal lymph nodes, and pleural effusion is rare. There are also a small number of patients with negative CT manifestations, which should be noted [4]; thus, the diagnosis of COVID-19 should not depend only on radiology.

**Figure 1:**
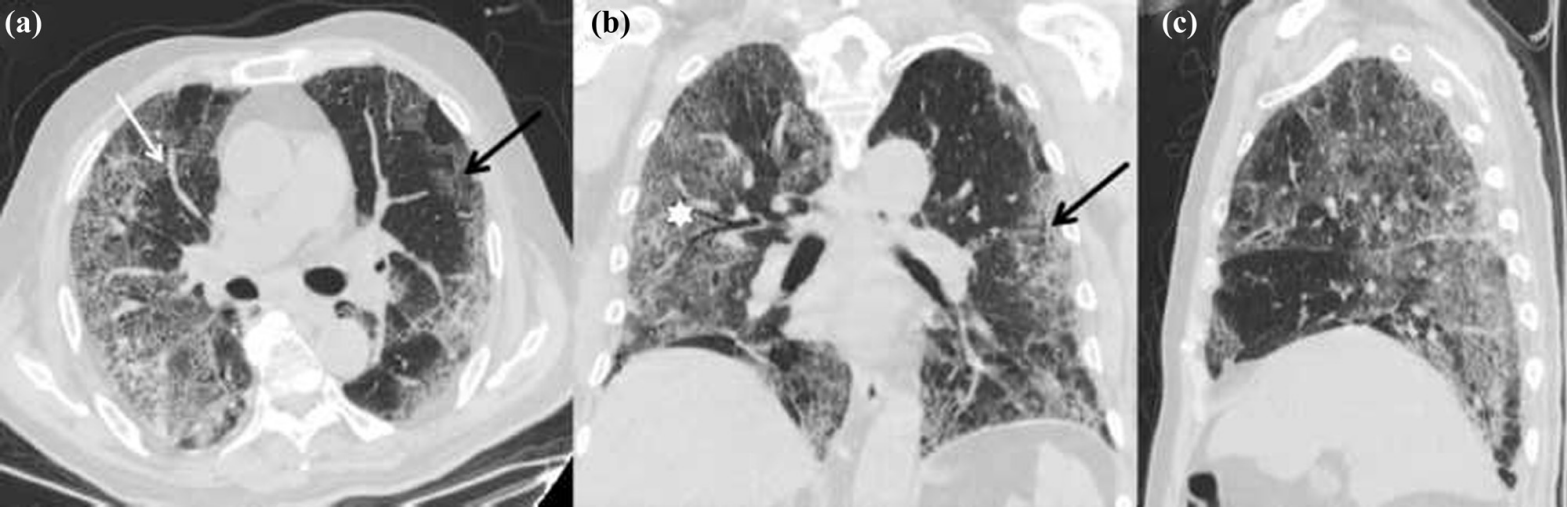
First CT scans of the patient (a: axial noncontrast CT image; b: coronal thin-section noncontrast CT image; and c: sagittal thin-section noncontrast CT image) showing large areas of GGO distributed mainly in subpleural (black arrows), thickened vascular shallow (white arrow), and bronchus shallow (white asterisk).

**Figure 2:**
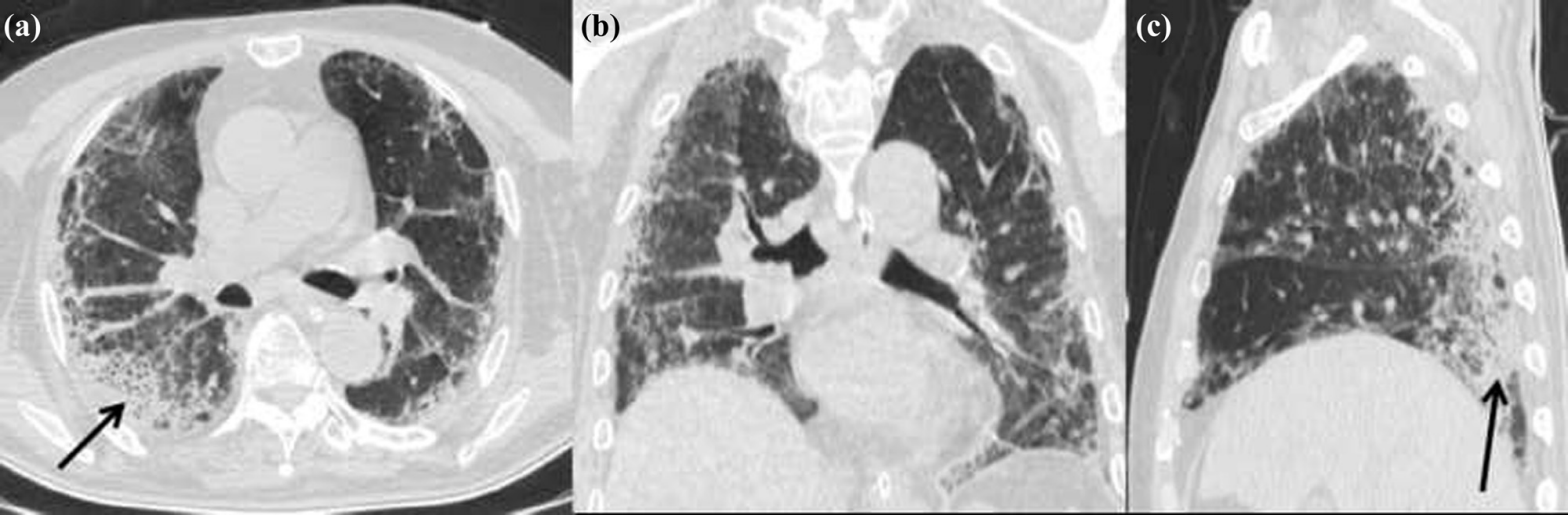
CT scans obtained 16 days after admission (a: axial non-contrast CT image; b: coronal thin-section noncontrast CT image; and c: sagittal thin-section noncontrast CT image) showing the range of lesions reduced compared with the previous images; partial patchy consolidation (black arrows).

**Figure 3:**
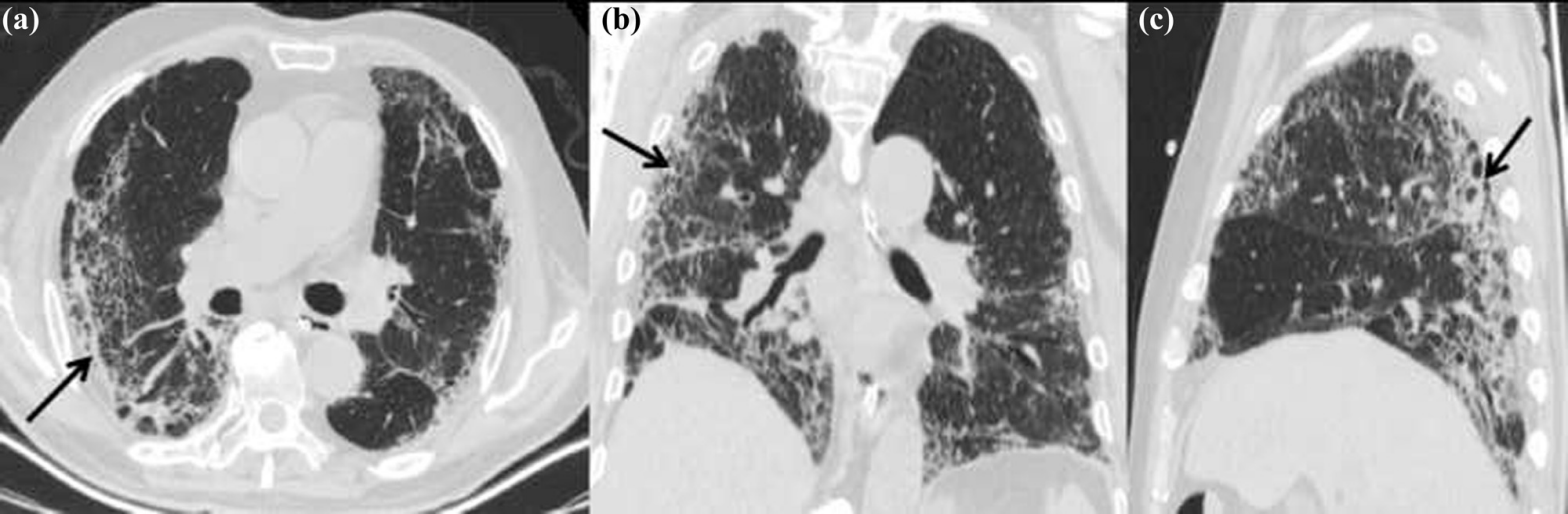
CT scans obtained 20 days after admission (a: axial non-contrast CT image; b: coronal thin-section non-contrast CT image; and c: sagittal thin-section noncontrast CT image) showing the range of lesions reduced significantly compared with previous images; fibrous changes in subpleural (black arrows).

**Figure 4:**
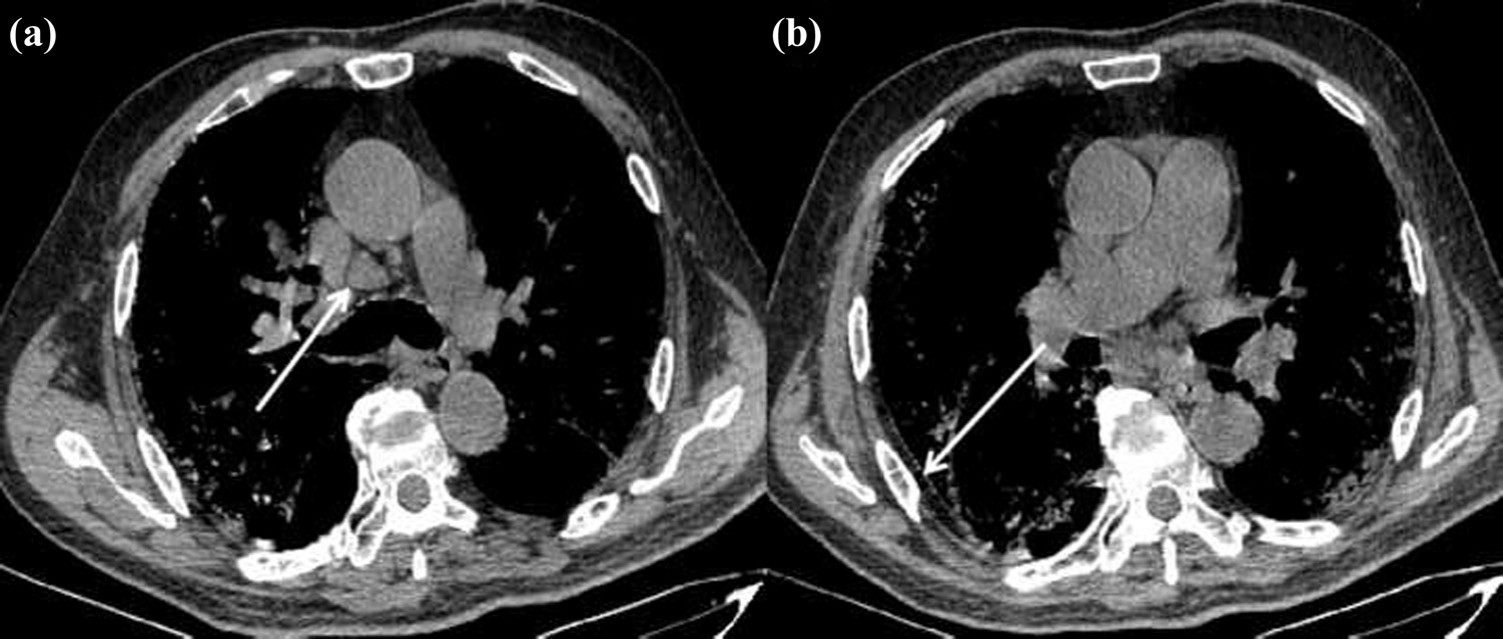
First CT scans (axial mediastinal noncontrast image) showing mediastinal enlarged lymph nodes (white arrow, a) and local thickening of the pleural (white arrow, b).

COVID-19 can be divided into four stages according to its imaging characteristics and lung involved: early stage, progressive stage, critical stage, and remission stage [3]. We report the case in a dynamic course. As the patient was admitted to the hospital in a critical stage, we did not obtain the classical imaging of early stage and progressive stage. However, we can see the recovery of the patient by CT imaging. The areas of GGO reduced obviously as the lesions were absorbed. The fibrous changes emerged later (Figures 1–4).

According to the related epidemiological history of the patients, the typical symptoms such as fever, dry cough, and fatigue, combined with the imaging manifestations of typical viral pneumonia and the results of laboratory examination, can make a preliminary diagnosis. The final diagnosis needs to be tested by the viral nucleic acid. If it is positive, the final diagnosis can be made.
